# Clinical Uses of Inhaled Antifungals for Invasive Pulmonary Fungal Disease: Promises and Challenges

**DOI:** 10.3390/jof9040464

**Published:** 2023-04-12

**Authors:** Nancy N. Vuong, Danielle Hammond, Dimitrios P. Kontoyiannis

**Affiliations:** 1 Division of Pharmacy, The University of Texas MD Anderson Cancer Center, Houston, TX 77030, USA; 2 Department of Leukemia, The University of Texas MD Anderson Cancer Center, Houston, TX 77030, USA; 3 Department of Infectious Disease, Division of Internal Medicine, The University of Texas MD Anderson Cancer Center, Houston, TX 77030, USA

**Keywords:** inhaled, antifungal, amphotericin B, fungal pneumonia, aspergillosis

## Abstract

The role of inhaled antifungals for prophylaxis and treatment of invasive fungal pneumonias remains undefined. Herein we summarize recent clinically relevant literature in high-risk groups such as neutropenic hematology patients, including those undergoing stem cell transplant, lung and other solid transplant recipients, and those with sequential mold lung infections secondary to viral pneumonias. Although there are several limitations of the available data, inhaled liposomal amphotericin B administered 12.5 mg twice weekly could be an alternative method of prophylaxis in neutropenic populations at high risk for invasive fungal pneumonia where systemic triazoles are not tolerated. In addition, inhaled amphotericin B has been commonly used as prophylaxis, pre-emptive, or targeted therapy for lung transplant recipients but is considered as a secondary alternative for other solid organ transplant recipients. Inhaled amphotericin B seems promising as prophylaxis in fungal pneumonias secondary to viral pneumonias, influenza, and SARS CoV-2. Data remain limited for inhaled amphotericin for adjunct treatment, but the utility is feasible.

## 1. Introduction and Background

Inhalation of ubiquitously present fungal conidia is an everyday phenomenon and not surprisingly, invasive fungal disease commonly manifests as pneumonia. Fungal pneumonia is most commonly caused by opportunistic molds, *Cryptococcus*, and geographically restricted dimorphic fungi [1]. These acute, subacute, or chronic lung fungal infections result in significant morbidity, and along with disseminated disease are associated with the highest mortality [2,3]. Despite tremendous improvement in supportive care and systemic antifungal prophylaxis, these infections continue to constitute a formidable therapeutic challenge for an ever-expanding population of patients with severe and protracted immunosuppression. Patients treated with a variety of immunosuppressive treatments for malignant or autoimmune diseases are frequently affected and as of lately, sequential lung mold infections are commonly encountered due to an increasing population of patients with severe viral pneumonia such as influenza or SARS CoV2 [4,5,6].

Because of the tremendous burden of opportunistic fungal pneumonias, antifungal prophylaxis with oral triazoles has become a standard of care in high-risk patients, especially those with acute leukemia or transplant recipients. This strategy has been shown to be effective and reasonably well tolerated. However, despite their success, oral triazoles can be problematic due to significant drug–drug interactions, suboptimal pharmacokinetics, and acute, subacute, or chronic toxicities [7,8]. These considerations have sparked interest in the use of alternative administration of antifungals via aerosolization as prophylaxis and as adjunct treatment in select cases of fungal pneumonia in high-risk patients. In this review, we aim to provide a brief overview of the conundrums in defining a “space” for inhaled antifungals in the context of our current antifungal armamentarium which is comprised with 12 FDA-approved systemic antifungals. We will also critically review the limited literature of the various antifungal drugs, mainly amphotericin B, used as inhalation in randomized or open labelled prospective studies and discuss potential prospects of the inhalation approach in the future as prophylaxis and treatment in the populations at most risk for invasive pulmonary fungal disease.

## 2. Methods

We used PubMed Medline to search for the currently published literature on the clinical uses of inhaled antifungal agents for invasive pulmonary fungal infections. Our search included articles in English and studies in humans of any age. We used the following list of keywords in our search query: inhale(d), aerosol(ized), nebulize(d), antifungal, -azole, itraconazole, isavuconazole, isavuconazonium, fluconazole, posaconazole, voriconazole, echinocandin, anidulafungin, micafungin, caspofungin, polyene, amphotericin B, and invasive pulmonary fungal infection. Additional articles of interest were also reviewed for inclusion. Articles published from 1960–2022 were reviewed, but only those published from 2002–2022 are included. The following was excluded from our review: topical instillation, interventional bronchoscopic treatment, pentamidine, cyclosporin, infections due to *Pneumocystis jirovecii,* case reports, allergic bronchopulmonary aspergillosis, aspergillomas and fungal asthma as these entities do not pathophysiologically belong to invasive fungal pneumonias.

## 3. Problems in Interpreting Inhaled Antifungal Literature

The literature on the use of inhaled antifungals in high-risk patients is limited. With two exceptions in neutropenic and lung transplant patients, respectively [9,10], there are no randomized studies in that area. Available studies are hard to compare, as study designs used different doses or dosing intervals, different delivery systems, frequently enrolled a mixed population at risk (leukemia, stem cell transplant, solid organ transplant, intensive care unit), spanned over several decades, and commonly used historical controls. Typically, most of these studies are underpowered, single center, have variable follow up, and frequently applied different neutrophil thresholds for enrollment of neutropenic patients and different criteria for diagnosing proven of probable pneumonia. Thus, not surprisingly, the background incidence of the main targeted pulmonary mycosis, invasive pulmonary aspergillosis (IPA), differs among studies, adding another layer of difficulty and complexity in cross- study evaluation. Furthermore, formal cost-effectiveness analysis and details regarding discontinuation data, logistical challenges (e.g., technical issues with the nebulizers), and compliance difficulties are lacking. In addition, most of the studies did not stratify regarding status of underlying malignant disease (e.g., during remission induction, refractory/relapse leukemia, or underlying stem cell transplant, and whether patients were housed in HEPA filtered rooms). Finally, concomitant systemic antifungal therapy is reported for most but not all studies.

Amphotericin B (AMB)-based formulations were the predominant antifungal drug used in such studies. All the studies had a primary prophylaxis scope to prevent the development of IPA. Importantly, there are no head-to-head comparison studies on inhaled antifungals (i.e., AMB) versus other systemic mold-active antifungals commonly used for primary prophylaxis such as triazoles (i.e., posaconazole, voriconazole, or isavuconazole). Outside prophylaxis studies, there are only case series and case reports on the use of inhaled antifungals as adjunct therapies in refractory cavitary mold pneumonia reporting promising results, but which might be inherently subject to publication biases and confounding issues. Table 1 and Table 2 show the published clinical studies using nebulized AMB as prophylaxis in patients with hematological malignancies and lung transplant recipients, respectively. Because of the heterogeneity, the aforementioned limitations and lack of good quality studies, there has not been a consensus on the role of inhaled AMB as a primary mode for mold-active prophylaxis by the different societies such as the Infectious Diseases Society of America (IDSA), the European Conference on Infections in Leukaemia(ECIL), the International Society for Heaty & Lung Transplantation (ISHLT) or the European Society of Clinical Microbiology and Infectious Diseases (ESCMID) [11,12,13,14,15,16]. Based on the single available randomized placebo-controlled study in neutropenic patients with hematologic malignancy, an inhaled liposomal AMB dosed at 12.5 mg twice weekly appears to be the most optimal regimen that has reasonable tolerability and potential efficacy [10]. In addition, the use of an inhaled AMB-based strategy seems appealing in patients undergoing lung transplantation and is frequently used as prophylaxis and adjunct treatment of IPA [17]. Specifically, inhaled AMB is a common practice because of the unique pathophysiological features of IPA in lung transplants where airway disease and anastomotic stump Aspergillus tracheobronchitis are common and amenable to local antifungal delivery [18]. Thus, inhaled AMB is a recommended strategy as universal prophylaxis or preemptive therapy and as adjunct treatment for IPA in lung transplant recipients [12,15,19]. In contrast, the published experience of inhaled AMB outside lung transplant in other solid organ transplant (SOT) recipients (e.g., heart and liver) is scarce and is limited to small non-randomized trials, case series, and meta-analyses [9,20,21,22,23]. Consensus from transplant societies (ESCMID, European Confederation of Medical Mycology (ECMM), European Respiratory Society (ERS), the American Society of Transplantation-Infectious Disease Community of Practice (AST-IDCOP), ISHLT) differ and the role also appears limited in the era of broad azole prophylaxis and risk stratification [13,15,16].

## 4. Inhaled AMB as Treatment in Patients with Fungal Pneumonia

There are only two retrospective, single institution case series, indicating a potential adjunct role of inhaled AMB in hematological malignancies, including those undergoing stem cell transplant, for the treatment of fungal pneumonia.

In 2013, Safdar and Rodriguez evaluated the efficacy and safety of aerosolized ABLC as an adjunct treatment for fungal lung disease. This retrospective study identified 32 immunosuppressed adult patients that received aerosolized ABLC 50 mg twice daily with concurrent systemic antifungal therapy. Probable or proven fungal lung pneumonia was documented in 13 patients. Clinical and radiographic resolution occurred in 16 patients (50%). Treatment with aerosolized ABLC was tolerated without serious toxicity [30].

In 2019, Venanzi et al. evaluated the efficacy and tolerability of systemic antifungal therapy with and without aerosolized lipid AMB (10 received ABLC and 1 patient received L-AMB) in cases of probable or proven IPA. This was a single-center retrospective cohort study. Patients with proven or probable IPA were started on systemic antifungal therapy (SAT) with or without aerosolized lipid AMB for 4 weeks. Patients who were started on aerosolized lipid AMB after the 4 weeks of SAT were considered secondary prophylaxis. Patients with hematological and solid tumor malignancies, solid organ transplant recipients, and severe chronic obstructive pulmonary disease were included. A total of 33 patients were included for analysis, 22 in the SAT only group and 11 that received aerosolized treatment (5 as adjunct and 6 as secondary prophylaxis). Clinical outcomes at 3 months were better for those receiving aerosolized lipid AMB, but this was not significant. Reduced mortality was seen at 12 months after multivariate analysis (HR 0.258; 95% CI 0.072–0.922; *p* = 0.037) [31].

## 5. Inhaled AMB as Treatment or Prophylaxis in Patients with Post-Viral Fungal Pneumonia

IPA has been reported to complicate severe respiratory infections from influenza and lately SARS CoV-2 (COVID-19) [32]. The incidence of influenza-associated aspergillosis (IAA) is considerably variable based on the geographical location but is documented in up to 14% in patients without the typical immune suppressive host factors previously mentioned [4,33,34,35,36,37,38]. Although the definition and classification criteria for the diagnosis of COVID-19 associated pulmonary aspergillosis (CAPA) remains uncertain, it has been reported that 1.6 to 38% of patients with severe COVID-19 in the intensive care unit develop CAPA [33,39,40]. Similarities in risk factors with IAA prompted concerns for IPA that emerged early in the COVID-19 pandemic [41]. Systemic antifungal therapy remains the standard of care for the management of post-viral IPA, with the potential use of adjunct aerosolized AMB as an option [5,42,43,44,45]. This might be especially important for post influenza aspergillosis, as Aspergillus tracheobronchitis is a common clinical manifestation of post influenza aspergillosis [46].

There are only four small studies on prophylactic inhalation of AMB showing promising results for those at high risk of CAPA [6,47,48,49]. Table 3 summarizes the available studies of inhaled amphotericin B as prophylaxis. Literature using inhaled antifungals for both IAA and CAPA as either treatment or prophylaxis are limited and consensus based on expert opinions have not been established [12,42,50].

## 6. Inhaled Antifungals under Clinical Testing

Outside AMB-based inhalation formulations, nebulization of azoles (voriconazole, itraconazole, posaconazole) have been tried with disappointing results, as these drugs have a very rapid elimination and swift systemic absorption, both in animal models and in a limited number of human subjects [51]. The use of inhaled echinocandins is theoretically possible but there are very limited clinical data [51]. The spectrum of echinocandins is limited and its lack of activity against non-Aspergillus molds makes them less suitable for development as an inhaled formulation. In contrast, there is promise for the use of opelconazole (PC945) as a nebulized suspension [52]. Opelconazole is a novel broad spectrum triazole antifungal that was administered in animal models via nebulization and showed a long duration of action in the lung. It only achieves very low systemic plasma concentrations and thus is devoid of systemic drug-drug interactions [52]. In preclinical testing, the antifungal effects of opelconazole against *Aspergillus fumigatus* accumulate on repeat dosing and the inhaled drug synergizes when it is given in combination with various systemic antifungal agents [52]. Opelconazole was well tolerated in heathy volunteers and patients with asthma, and as with preclinical models, a long lung residency time and minimal systemic absorption was seen [52]. Anecdotal evidence from cases where inhaled opelconazole was used in IPA unresponsive to systemic antifungals show the agents appear to work [52]. A phase three trial investigating the safety and efficacy of inhaled opelconazole in combination with other antifungal therapy for the treatment of refractory IPA is under way [53]. Opelconazole might also have promise as prophylaxis in patients with cystic fibrosis, in lung transplant recipients and in other patients with chronic aspergillosis lung diseases. A clinical program to study opelconazole in these contexts is being developed [54].

## 7. Perspectives

In view of the significant biochemical, pharmacologic, and manufacturing challenges along with challenges for preclinical validations, there is currently no perfect inhaled antifungal. Table 4 depicts the key characteristics of an optimal inhaled antifungal product and some difficulties in preclinical validation. Regarding existing inhaled antifungals, only AMB based formulations were tested and available literature is suboptimal to allow firm conclusions. It seems that inhaled liposomal AMB could be an alternative method of prophylaxis in selected high-risk neutropenic populations where systemic triazoles are not tolerated, although it remains unclear whether that subgroup of patients is also in need for protection by non-azole based systemic prophylaxis with parenteral echinocandins. In addition, inhaled AMB has been commonly used as prophylaxis, pre-emptive or targeted therapy for lung transplant recipients but is considered a secondary alternative for other solid organ transplant recipients. Whether these patients would benefit from systemic prophylaxis against non-mold fungal infections, specifically Candida, remains uncertain and patient-level risk stratification is paramount. Finally, although data are limited, inhaled AMB seems promising as prophylaxis in fungal pneumonias secondary to viral pneumonias, influenza, and SARS CoV-2 in patients that are mechanically ventilated.

The recent developments with the emergence of resistance of fungi to azoles and the shift to more targeted therapies for the treatment of hematologic cancer might force us to reevaluate the merit of inhaled antifungals [55]. The emergence of azole resistance in *Aspergillus*, partially driven by the widespread agricultural used of azoles [56], has devitalized to some extent the potency of triazoles. Theoretically, the local delivery of a high concentration fungicidal drug such as AMB in the lungs would result in less selection pressure for antifungal resistance, although this concept has not been studied. Furthermore, the explosion of molecularly active therapies with which azoles have significant drug–drug interactions, the improvement of non-culture based diagnostic methods that allow early therapy, and the expansion of the antifungal armamentarium with new first in class antifungals might revitalize the interest in using inhaled antifungals [57,58,59,60,61]. However, in view of the introduction to clinical testing of antifungals with novel in mechanism of action, it remains to be seen if those agents that are devoid of the problem of azole resistance and drug–drug interactions and which allow systemic protection might be better options for prophylaxis, thus still leaving inhaled antifungals as second options [62]. Finally, the role of promising non-AMB based inhaled antifungals, such as PC945 (opelconazole), currently undergoing clinical testing awaits further validation. Table 5 lists some of the challenges and questions in future preclinical and clinical studies in the field of inhaled antifungals.

## Figures and Tables

**Table 1:** Inhaled AMB as prophylaxis in patients with hematological malignancies/undergoing stem cell transplant.

Reference	Type of Study	Study Population	Formulation and Delivery System	Dosage and Duration	Concomitant Antifungal	Outcome	Side Effects	Comments
2006 Alexander et al. [24]	Prospective, open-label non-comparative study assessing safety and tolerability	Allogeneic stem cell transplant *n* = 40	Aerosolized ABLC	50 mg daily × 4 days, then once per week for 13 weeks (total 17 doses)	Fluconazole daily as prophylaxis through transplant day 100	3 cases of proven IFI, of which 1 developed while on treatment	Safe and well-tolerated Cough, nausea, taste disturbance or vomiting in 2.2%	
2008 Rijnder et al. [10]	Randomized, double-blind placebo-controlled trial	Neutropenic patients with hematologic malignancies, neutropenia expected >10 days *n* = 271	Nebulized L-AMB vs. Placebo	12.5 mg nebulized twice weekly until neutrophils above 300 cells/mm^3^	All patients received prophylactic fluconazole	Developed IPA (ITT): L-AMB 6/139 vs. Placebo 18/132 (OR 0.26; 95%CI, 0.09–0.72; *p* = 0.005)	Some, but none serious Cough: L-AMB 16 vs. Placebo 1 (*p* = 0.002)	On-treatment analysis: L-AMB 2/91 vs. Placebo 13/97 (OR 0.14; 95% CI, 0.02–0.66; *p* = 0.007)
2011 Hullard-Pulstinger et al. [25]	Prospective phase II trial, an evaluation of toxicity vs. historical control (*n* = 105)	Patients expected to be neutropenic >10 days after chemotherapy or stem cell transplant Treatment *n* = 98	Nebulized L-AMB	12.5 mg for 4 consecutive days, then twice weekly until neutrophil recovery (>500 cells/mm^3^)	Fluconazole prophylaxis allowed and used in majority of patients	Unable to show reduction in IFI, early termination of trial	41 patients terminated trial early due to unpleasant treatment experiences, not toxicities	Voriconazole was available for intervention group but not for control group
2012 Nihtinen et al. [26]	Retrospective, single center study with historical control evaluating inhaled AMB as prophylaxis Vs. historical control (*n* = 257)	Stem cell transplant patients (acute GvHD treated with high-dose methylprednisolone) *n* = 357	Nebulized AMB-d	25 mg daily for 2 or 3 months, per attending	Systemic antifungal prophylaxis not routinely used in either group	Significantly more patients in control group had detectable IPA 17/257 (6.6%) vs. Prophylaxis group 9/354 (2.5%) *p* = 0.007	Prophylaxis was well tolerated	
2015 Chong et al. [27]	Prospective cohort evaluation of the efficacy and cost effectiveness of aerosolized L-AMB	AML patients *n* = 127	Nebulized L-AMB	12.5 mg twice a week at beginning of first and second cycle of chemotherapy, continued until recovery of neutrophils (2 consecutive counts of ≥0.2 × 10^9^ L^−1^ or one ≥0.5 × 10^9^ L^−1^)	Prophylaxis with fluconazole	Incidence of IPA during the first and second chemotherapy cycles was 9.5% and was a significant decrease when compared to the control group (23.4%), *p* = 0.0064		Prophylaxis with inhaled L-AMB stopped during auto or allogeneic stem cell transplant

AMB: amphotericin; AMB-d: amphotericin B deoxycholate; ABLC: amphotericin B lipid complex; CI: confidence interval; L-AMB: liposomal amphotericin; AML: acute myeloid leukemia; GvHD: graft versus host disease; IPA: invasive pulmonary *Aspergillosis*; IFI: invasive fungal infection; ITT: intention to treat; OR: odds ratio.

**Table 2:** Inhaled AMB as prophylaxis in lung transplant recipients.

Reference	Type of Study	Study Population	Formulation and Delivery System	Dosage and Duration	Concomitant Prophylactic Antifungal	Outcome	Side Effects	Comments
2002 Minari et al. [28]	Retrospective study with historical control (10-year study) of universal Aspergillus prophylaxis in lung transplant	Lung transplant recipients *n* = 183	Aerosolized AMB-d	5–10 mg twice daily, immediately post-transplant for up to two weeks	Once oral intake tolerated, patients converted to itraconazole	24 patients diagnosed with IPA All had lung involvement with an incidence of 40.5/1000patient-years and overall mortality of 50% The incidence of IA was significantly higher in a historical control group (49.7/1000patient-years vs. 31.6/1000 patient-years, *p* ≤ 0.05)		Advocate using aerosolized AMB followed by itraconazole as prophylaxis in lung transplant recipients
2004 Drew et al. [9]	Prospective, randomized, double-blind study comparing safety and tolerability of AMB-d and ABLC	Lung transplant recipients *n* = 100 Randomized 1:1	Aerosolized AMB-d Or Aerosolized ABLC	AMB-dd 25 mg Or ABLC 50 mg Once daily for four days, then once weekly for 7 weeks	Nonabsorbable antifungal agent (nystatin) permitted	Primary prophylaxis failure was similar in both groups (14.3% AMB-d vs. 11.8% ABLC) with *Aspergillus* infections documented in only 2 patients No fungal pneumonias were observed in either group	Adverse events more common in AMB-d	
2010 Monforte et al. [29]	Comparative, prospective observational study with historical control on the feasibility, tolerability, and outcomes of nebulized amphotericin	Lung transplant recipients *n* = 104 L-AMB Historical control: *n* = 49 AMB-d	Nebulized L-AMB vs. AMB-d	L-AMB 25 mg three times weekly for 60 days post-transplant, continued at 25 mg once weekly on days 60–180, then 25 mg once every two weeks thereafter AMB-d 6 mg every 8 h immediately post-transplant for 120 days, then 6mg once daily for life	Not stated	Development of IPA: L-AMB 2/104 (1.9%) vs. AMB-d historical control 2/49 4.1% *p* = 0.43	Well tolerated	

AMB: amphotericin B; AMB-d: amphotericin B deoxycholate; ABLC: amphotericin B lipid complex; L-AMB: liposomal amphotericin; IPA: invasive pulmonary *Aspergillosis*; IFI: invasive fungal infection.

**Table 3:** Inhaled AMB as prophylaxis for IPA following viral pneumonia.

Reference	Type of Study	Study Population	Formulation and Delivery System	Dosage and Duration	Outcome	Side Effects	Comments
2020 Rutsaert et al. [47]	Prospective study of L-AMB as prophylaxis after raised suspicion of IPA in COVID-19 patients	All mechanically ventilated COVID-19 patients, sample size not defined	Nebulized L-AMB	12.5 mg, duration not specified	No new cases of IPA identified after initiation of prophylaxis		Environmental sources ruled out by air sampling. All mechanically ventilated patients screened with serum galactomannan twice weekly
2021 Van Ackerbroeck et al. [6]	Retrospective observational comparison of L-AMB for prophylaxis of CAPA	Mechanically ventilated *n* = 32 received L-AMB *n* = 18 no prophylaxis	Nebulized L-AMB	12.5 mg twice a week, duration not specified	Development of CAPA/AT occurred in 11 patients that did not receive prophylaxis compared to 3 that did (risk ratio 0.15, 95%CI 0.05 to 0.48, *p* < 0.001) Further development of Aspergillus colonization in endotracheal aspirates was significantly lower in the prophylaxis group (risk ratio 0.28, 95%CI 0.10 to 0.81, *p* = 0.017)	Well-tolerated	
2022 Soriano et al. [49]	Prospective observational cohort study evaluating use of inhaled ABLC	All mechanically ventilated patients *n* = 45	Inhaled ABLC	50 mg every 48 h, duration not specified	None developed CAPA	Well-tolerated 8.8% bronchospasm 33.3% with drug buildup in ventilator	Surveillance protocol for CAPA in mechanically ventilated COVID-19 patients identified an outbreak
2022 Melchers et al. [48]	Retrospective cohort study evaluating use of nebulized AMB-d as prophylaxis in mechanically ventilated ICU patients with COVID-19	Mechanically ventilated ICU patients with COVID-19 *n* = 39 *n* = 16 nebulized AMB-d	Nebulized AMB-d	20 mg in two or four divided doses	Incidence of positive Aspergillus cultures, positive BAL serological markers, and tracheobronchial lesions was significantly lower in the prophylaxis group compared to the control (9% vs. 53%, 20% vs. 60%, and 9% vs. 47%), respectively No observed cases of proven CAPA were seen in the prophylaxis group Overall frequency of probable or proven CAPA was much lower in the prophylaxis group Overall mortality at 90-days was 21% and was similar in both groups		

AMB: amphotericin B; AMB-d: amphotericin B deoxycholate; ABLC: amphotericin B lipid complex; AT: Aspergillus tracheobronchitis; BAL: bronchoalveolar lavage; CAPA: COVID-19 associated pulmonary *Aspergillosis*; COVID-19: coronavirus disease 2019; L-AMB: liposomal amphotericin; IPA: invasive pulmonary *Aspergillosis.*

**Table 4:** Clinical characteristics of an ideal inhaled antifungal.

Slow absorption from the lungs and minimal systemic absorption No systemic toxicity No drug–drug interactions
High protein plasma binding Minimization of systemic free drug concentration
Sustained local concentrations in the airways Increased antifungal efficacy Less likelihood of acquisition of resistance
High lung residence times Longer duration of action → No need for high doses or frequent administration Accumulation on repeat dosing → extended prophylactic effect
Small antifungal drug particles (e.g., <5 mm) Deposition to distal airways
Limited interference with mechanical ventilation machinery
Delivery system (e.g., nebulizers) that is convenient, portable, easy to operate, has low cost
Broad spectrum of activity against respiratory fungal pathogens
Activity against fungal biofilms
Intracellular accumulation within lung neutrophils and macrophages
Synergy with systemically administered antifungals and effector immune cells in lungs
No local respiratory side effects (e.g., bronchospasm, metallic taste, breathlessness, decrease in PFTs) *
Proof of principle demonstration of safety and activity in in vitro and preclinical in vivo studies

* Including in patients with underlying lung diseases (e.g., asthma); PFTs: pulmonary function tests.

**Table 5:** Some ongoing and future questions regarding inhaled antifungals.

Preclinical/Translational
What are the optimal physicochemical properties (e.g., size, lipophilicity, solubility) of an inhaled antifungal?
Since an inhaled drug achieves a concentration gradient in the airways (central > distal airways), is there an increased likelihood of resistance?
Is a microbiological end point predictive of a meaningful clinical endpoint? Is a decrease of the burden more realistic than fungal eradication?
What is the role of fungal biomarkers (e.g., Aspergillus GM) in BAL and/or serum for fungal load assessment?
How do in vitro studies (e.g., bronchial epithelial cell lines) predict in vivo behavior and toxicity potential of an inhaled antifungal?
Due to differences in anatomy, physiology and local immunology, how useful are mouse models of infections using inhaled antifungals?
How to best estimate relevant PKs of inhaled antifungals in normal and infected human lungs? Role of NMR spectroscopy, PET, compound concentration in sputum, BAL, or epithelial lining fluid (or combinations)?
**Clinical**
Are inhaled antifungals best used as adjunct treatment only for central airway or cavitary disease?
Are inhaled antifungals best used as primary prophylaxis/? Is there a role for secondary prophylaxis?
For lipid formulations of antifungals (e.g., liposomal AMB), is there a concern of fatty infiltration and foamy macrophage accumulation in the lungs?
Do inhaled antifungals work less well in the setting of excess mucus (e.g., COPD, bronchiectasis) or biofilms (e.g., CF)?
In addition to AMB, what is the potential for aerosolized echinocandins, azoles or other antifungal drugs?
What is the impact of aerosolized antifungals on the treatment of fungal sinusitis?
What is the optimal delivery technology to deliver intact drugs to the distal airways?
What is the optimal regulatory pathway for the development and approval of an inhaled antifungal? What is an optimal trial design, end points and scenarios for use?
As a portion of inhaled antifungals might be swallowed, how does one evaluate the impact of oral absorption, when orally active compounds are repurposed for delivery via the inhalation route?

GM: galactomannan; BAL: bronchoalveolar lavage, NMR: Nuclear Magnetic Resonance; PET: Positron Emission Tomography; COPD: Chronic Obstructive Pulmonary Diseases; CF: Cystic Fibrosis; PK: pharmacokinetics; AMB: Amphotericin B.

## Data Availability

Not applicable.
